# Genetic Variations in the Flanking Regions of *miR-101-2* Are Associated with Increased Risk of Breast Cancer

**DOI:** 10.1371/journal.pone.0086319

**Published:** 2014-01-24

**Authors:** Jiaping Chen, Zhenzhen Qin, Yue Jiang, Yanru Wang, Yisha He, Juncheng Dai, Guangfu Jin, Hongxia Ma, Zhibin Hu, Yongmei Yin, Hongbing Shen

**Affiliations:** 1 MOE Key Laboratory of Modern Toxicology, School of Public Health, Nanjing Medical University, Nanjing, China; 2 State Key Laboratory of Reproductive Medicine, Institute of Toxicology, Nanjing Medical University, Nanjing, China; 3 Section of Clinical Epidemiology, Jiangsu Key Lab of Cancer Biomarkers, Prevention and Treatment, Cancer Center, Nanjing Medical University, Nanjing, China; 4 Department of Medical Oncology, Jinling Hospital, Southern Medical University, Nanjing, China; 5 Department of General Surgery, The First Affiliated Hospital of Nanjing Medical University, Nanjing, China; MOE Key Laboratory of Environment and Health, School of Public Health, Tongji Medical College, Huazhong University of Science and Technology, China

## Abstract

Genetic variants in human microRNA (miRNA) genes may alter mature miRNA processing and/or target selection, and likely contribute to cancer susceptibility and disease progression. Previous studies have suggested that *miR-101* may play important roles in the development of cancer by regulating key tumor-associated genes. However, the role of single nucleotide polymorphisms (SNPs) of *miR-101* in breast cancer susceptibility remains unclear. In this study, we genotyped 11 SNPs of the *miR-101* genes (including *miR-101-1* and *miR-101-2*) in a case-control study of 1064 breast cancer cases and 1073 cancer-free controls. The results revealed that rs462480 and rs1053872 in the flank regions of *pre-miR-101-2* were significantly associated with increased risk of breast cancer (rs462480 AC/CC *vs* AA: adjusted OR = 1.182, 95% CI: 1.030–1.357, *P = *0.017; rs1053872 CG/GG *vs* CC: adjusted OR = 1.179, 95% CI: 1.040–1.337, *P = *0.010). However, the remaining 9 SNPs were not significantly associated with risk of breast cancer. Additionally, combined analysis of the two high-risk SNPs revealed that subjects carrying the variant genotypes of rs462480 and rs1053872 had increased risk of breast cancer in a dose-response manner (*P*_trend_ = 0.002). Compared with individuals with “0–1” risk allele, those carrying “2–4” risk alleles had 1.29-fold risk of breast cancer. In conclusion, these findings suggested that the SNPs rs462480 and rs1053872 residing in *miR-101-2* gene may have a solid impact on genetic susceptibility to breast cancer, which may improve our understanding of the potential contribution of miRNA SNPs to cancer pathogenesis.

## Introduction

Breast cancer is the leading cause of cancer-related deaths among women world-wide, with an estimated 1,383,500 new cases and 458,400 deaths in 2008. Although the incidence and mortality rates in developed countries have been decreasing during the past 25 years, both rates have been increasing in many developing countries [1]–[2]. In China, breast cancer is the most prevalent cancer and is ranked the sixth leading cause of death in Chinese women [3]. Except for ill-fitted environmental exposures, lifestyle and behavioral factors, many studies have also suggested that genetic factors are usually associated with the risk of breast cancer [4].

In recent years, the use of genome-wide association studies (GWAS) to screen disease-associated genetic variants, has led to the successful identification of numerous breast cancer susceptibility regions, supporting a polygenic model of breast cancer susceptibility [5]–[8]. However, such loci explain only a small percentage of the total risk, and important regions harboring genetic variants associated with breast cancer risk still remain to be identified.

MicroRNAs (miRNAs) are a class of small (∼22 nucleotides), non-coding single-stranded RNAs, which regulate gene expression by targeting mRNA for deregulation or translational repression [9]. At present, the biogenesis of miRNAs has been clearly described in mammals. In general, miRNA genes are initially transcribed by RNA polymerase II to form large, primary miRNAs (pri-miRNAs). These pri-miRNAs are subsequently cleaved into pre-miRNAs by Drosha and processed into a miRNA duplex of 19–22 nt, by the endonuclease enzyme Dicer. In most cases, only one strand of the miRNA duplex from either the 5′ or the 3′ arm of the pre-miRNA is selected as the mature miRNA and incorporated into the RNA-induced silencing complex (RISC) that can target specific protein-coding messenger RNA (mRNA) [9]–[10]. Recently, increasing evidence suggests that miRNAs regulate the expression of almost one-third of the human genome. Deregulation of mature miRNA expression has been demonstrated in many human cancers including breast cancer, and specific miRNAs have been used as markers to define molecular subtypes of various cancers [11]–[13].

Although the exact mechanisms underlying miRNA deregulation in cancer are not clear, the presence of single nucleotide polymorphisms (SNPs) in miRNA genes, including pri-miRNAs, pre-miRNAs and mature miRNAs, has been shown to influence the processing and/or target selection of miRNAs, thus affecting the risk of cancer [14]–[21]. For example, Hu *et al*. first reported that the rs11614913 SNP in the *miR-196a2* precursor was associated with survival of patients with non-small lung cancer (NSCLC) and risk of lung and breast cancer [15]–[17]. Further studies demonstrated that the rs11614913 SNP not only affected the mature processing of *miR-196a2*, but also influenced the interactions between *miR-196a2* and its downstream targets [15]. The functional relevance of the rs11614913 SNP in the *miR-196a2* precursor and breast cancer susceptibility was further confirmed by a research group at Yale University [18].

*MiR-101* belongs to a family of miRNAs involved in various cellular activities, including cell proliferation, invasion and apoptosis [22]. Genomic loci for *miR-101* have been identified on chromosome 1p31.3 (*miR-101-1*) and chromosome 9p24.1 (*miR-101-2*). *MiR-101* is frequently expressed at low levels in multiple malignancies including breast cancer, hepatocellular carcinoma, glioblastoma, prostate and gastric cancers [23]–[27]. Over-expression of *miR-101* has a tumor-suppressive effect in breast cancer, and *miR-101* has been shown to negatively regulate oncogenes including *EZH2* and *STMN1* [22], [27]. Furthermore, Sachdeva *et al*. reported that *miR-101* may promote MCF-7 cell growth in an estrogen-independent manner by enhancing AKT activation, suggesting a link between *miR-101* and estrogen-independent signaling in estrogen receptor (ER)-positive tumor cells [28]. However, to date, little is known about the role of *miR-101*-associated SNPs in breast cancer risk.

In this study, we hypothesized that polymorphisms of *miR-101* are associated with the susceptibility of breast cancer in women. To test this notion, we investigated the association of 11 SNPs located in the *miR-101* genes with breast cancer risk in a Chinese case-control study.

## Materials and Methods

### Ethics Statement

This case-control study was approved by the institutional review board of Nanjing Medical University. The design and performance of current study involving human subjects were clearly described in a research protocol. All participants were voluntary and would complete the informed consent in written before taking part in this research.

### Study Population

A total of 1064 breast cancer cases and 1073 cancer-free controls were included in this study, which has been described previously [29]. Patients with breast cancer were consecutively recruited between January 2004–April 2010 from the First Affiliated Hospital of Nanjing Medical University, Gulou Hospital and the Cancer Hospital of Jiangsu Province (Nanjing, China). Cancer-free controls were randomly selected from a cohort of more than 30,000 participants in a community-based screening program for non-infectious diseases conducted in the Jiangsu Province during the same period as breast cancer patients were recruited. Control subjects had no self-reported cancer history and were frequency matched to the breast cancer patients by age (±5 years) and residential areas (urban and rural). Information related to demographic data, menstrual and reproduction history and environment exposure history was obtained from each patient during a standardized interview, and 5 ml of venous blood was subsequently collected from each participant for genotyping assays. The estrogen receptor (ER) and progesterone receptor (PR) status of each patient was obtained from the hospital medical records.

### SNPs selection and genotyping

Tagging SNPs located within the vicinity of mature *miR-101-1* and *miR-101-2* genes (10 kB upstream and downstream) were selected using the International HapMap Project (http://www.hapmap.org), dbSNP (http://www.ncbi.nlm.nih.gov/-projects/SNP/) and UCSC (http://genome.ucsc.edu/) databases. The linkage disequilibrium value (r^2^<0.8) and minor allele frequency (MAF≥0.05) in the Chinese Han population (CHB) were further applied to screen SNPs. Based on this, 7 tagging SNPs (rs555146, rs578481, rs705509, rs7536540, rs1011210, rs12049119, rs489500) in the *miR-101-1* region and 7 tagging SNPs (rs462480, rs17718377, rs4742051, rs1537146, rs10974820, rs2236495, rs1053872) in *miR-101-2* region were selected. During the process of chip design, three SNPs (rs12049119, rs489500 and rs2236495) were excluded owing to the technical reasons. Therefore, there were totally 11 tagging SNPs were genotyped using the Illumina Infinium® BeadChip (Illumina Inc.) platform in all patient and control samples (n = 2137). Genotype calling was using the GenTrain version 1.0 clustering algorithm in GenomeStudio V2011.1 (Illumina). All SNPs were successfully genotyped with call rates >95%.

### Statistical analyses

Differences in the distributions of demographic characteristics, selected variables and genotypes frequencies between breast cancer cases and controls were analyzed by χ^2^ test and student *t* test. Associations between the genotypes and breast cancer risk were estimated by computing the odds ratios (ORs) and 95% confidence intervals (CIs) from logistic regression analyses. Adjustment factors for the associations included age, age at menarche and menopausal status. The Hardy–Weinberg equilibrium was tested by a goodness-of-fit *χ*2 test to compare the observed genotype frequencies to expected frequencies among the control subjects. All statistical analyses were performed with Statistical Analysis System software (version 9.1.3; SAS Institute, Cary, NC, USA).

## Results

Demographic information and variables for breast cancer patients (n = 1064) and controls (n = 1073) are presented in Table S1. The age of patients and controls was comparable after frequency-matching (*P*>0.05). Compared with control subjects, patients with breast cancer experienced an earlier menarche, later first live birth and a lower proportion of natural menopausal status(*P*<0.05). Of the 1064 patients with breast cancer, 490 (46.05%) were ER positive, while 506 (47.56%) were PR positive.

The chromosomal position and disease association of 11 SNPs are described in Table 1. The SNP rs1011210 deviated from Hardy–Weinberg equilibrium among controls (*P*<0.05) and was excluded from subsequent analyses. In multivariate logistic regression models, the rs462480 and rs1053872 residing in the 61 bp and 10 kb downstream of *pre-miR-101-2* were significantly associated with an increased risk of breast cancer, but not the remaining nine SNPs (Table 1).

**Table 1:** Summary of associations between 11 SNPs in *miR-101* and breast cancer risk.

SNP	Chr	Position	Location	Alleles^a^	Case^b^	Control^b^	Call rate	MAF^c^	HWE^d^	OR(95% CI)^e^	*P* value^e^
					N = 1064	N = 1073	(%)	(case/control)			
rs7536540	1p31.3	65,524,582	hsa-mir-101-1	G/C	338/528/197	351/510/211	97.94	0.434/0.435	0.291	0.998(0.881–1.131)	0.982
rs1011210	1p31.3	65,526,176	hsa-mir-101-1	A/G	405/516/133	442/466/159	97.19	0.371/0.367	0.048	–	–
rs555146	1p31.3	65,527,407	hsa-mir-101-1	C/A	838/205/20	832/218/23	97.94	0.115/0.123	0.064	0.930(0.772–1.121)	0.446
rs578481	1p31.3	65,528,755	hsa-mir-101-1	A/G	507/460/97	535/436/101	97.99	0.307/0.298	0.381	1.042(0.910–1.192)	0.552
rs705509	1p31.3	65,531,751	hsa-mir-101-1	G/A	326/512/216	339/510/220	97.33	0.448/0.444	0.293	1.034(0.914–1.171)	0.594
rs462480	9p24.1	4,850,436	hsa-mir-101-2	A/C	531/442/88	591/395/86	97.80	0.291/0.265	0.084	1.182(1.030–1.357)	0.017
rs17718377	9p24.1	4,854,472	hsa-mir-101-2	G/C	821/227/13	845/218/10	97.85	0.119/0.111	0.437	1.113(0.914–1.356)	0.287
rs4742051	9p24.1	4,858,997	hsa-mir-101-2	A/G	576/412/74	618/386/68	97.85	0.264/0.244	0.456	1.139(0.988–1.314)	0.074
rs1537146	9p24.1	4,859,303	hsa-mir-101-2	A/G	623/388/51	655/361/57	97.89	0.231/0.222	0.426	1.081(0.931–1.255)	0.308
rs10974820	9p24.1	4,859,872	hsa-mir-101-2	G/A	875/180/8	905/162/5	97.89	0.092/0.080	0.538	1.174(0.938–1.469)	0.162
rs1053872	9p24.1	4,860,643	hsa-mir-101-2	C/G	265/532/265	305/541/226	97.85	0.500/0.463	0.667	1.179(1.040–1.337)	0.010

a Major/minor allele;

b Major homozygote/heterozygote/rare homozygote between cases and controls;

c Minor allele frequency (MAF);

d *P* values for Hardy-Weiberger equilibrium (HWE) tests;

e Logistic regression with adjustment for age, age at menarche and menopausal status in additive model.

Furthermore, we conducted a joint analysis of these two SNPs rs462480 and rs1053872 (Table 2). There was a significant trend for the increased risk of breast cancer with the increasing number of variant genotypes (*P*_trend_ = 0.002). In the combined dataset, compared with subjects with “0–1” risk allele of the two SNPs, subjects carrying “2–4” risk alleles resulted in 1.29-fold (95% CI, 1.08–1.54; *P* = 0.005) increased risk of breast cancer.

**Table 2:** Cumulative effect of rs462480 and rs1053872 in the flanking region of *miR-101-2* on breast cancer risk.

No. of risk allele^a^	Cases	Controls	OR(95%CI)^b^	*P* b
	N (%)	N (%)		
0	251(23.70)	291(27.12)	1	
1	234(22.10)	258(24.04)	1.03(0.80–1.33)	0.815
2	356(33.62)	346(32.25)	1.22(0.97–1.54)	0.096
3–4	218(20.59)	178(16.59)	1.48(1.13–1.94)	0.005
Trend				0.002
Binary classification				
0–1	485(45.80)	549(51.20)	1	
2–4	574(54.20)	523(48.80)	1.29(1.08–1.54)	0.005

a The rs462480 C allele and rs1053872 G allele were assumed as risk alleles based on main effect of individual locus;

b Adjusted for age, age at menarche, menopausal status.

We also performed stratification analyses for the combined effect of rs462480 and rs1053872 based on age, age at menarche, first live birth, menopausal status, ER and PR status. As shown in Table 3, the increased breast cancer risk associated with “2–4” risk alleles of rs462480 and rs1053872 was significant among women with older age, earlier menarche, later first live birth, negative ER and PR and postmenopausal status, compared with subjects with the “0–1” risk allele. However, we did not observe any significant differences of these two SNPs in different strata (*P*>0.05 for heterogeneity tests).

**Table 3:** Stratification analysis on the association of rs462480 and rs1053872 in the flanking region of miR-101-2 with breast cancer risk.

Characteristics	Case N(%)		Control N(%)		OR(95%CI)^c^	*P* c	*P* d
	0^a^	1^b^	0^a^	1^b^			
Age							
<51	280(47.6)	308(52.4)	275(50.8)	266(49.2)	1.19(0.93,1.52)	0.16	0.317
≥51	205(43.5)	266(56.5)	274(51.6)	257(48.4)	1.43(1.10,1.86)	0.006	
Menopausal status							
Premenopausal	242(47.3)	270(52.7)	255(50.6)	249(49.4)	1.14(0.89,1.47)	0.305	0.148
Postmenopausal	193(42.9)	257(57.1)	275(52.5)	249(47.5)	1.49(1.15,1.94)	0.003	
Age at menarche							
<16	272(45.5)	326(54.5)	224(54.5)	187(45.5)	1.43(1.11,1.85)	0.005	0.225
≥16	205(46.3)	238(53.7)	323(49.0)	336(51.0)	1.15(0.90,1.46)	0.275	
Age at first live birth							
<24	116(48.5)	123(51.5)	184(49.7)	186(50.3)	1.09(0.78,1.51)	0.617	0.259
≥24	339(45.0)	415(55.0)	351(52.3)	320(47.7)	1.37(1.10,1.71)	0.005	
ER status							
Positive	234(48.1)	253(52.0)			1.17(0.94,1.46)	0.17	0.557
Negative	173(45.9)	204(54.1)			1.29(1.02,1.65)	0.038	
PR status							
Positive	245(48.6)	259(51.4)			1.14(0.92,1.42)	0.24	0.357
Negative	162(45.0)	198(55.0)			1.33(1.04,1.70)	0.024	

a Subjects with 0–1 risk allele of rs462480 and rs1053872;

b Subjects with 2–4 risk alleles of rs462480 and rs1053872;

c Derived from logistic regression with an adjustment for age, age at menarche and menopausal status;

d *P* for heterogeneity test.

## Discussion

In this study, we evaluated the association of 11 tagging SNPs located in the *miR-101* gene and predisposition to breast cancer in a case-control study. We found that rs462480 and rs1053872 in the flank region of *pre-miR-101-2* were significantly associated with the increased risk of breast cancer in the Chinese population. To our knowledge, this is the first study to evaluate the association of *miR-101*-related polymorphisms and breast cancer susceptibility.

*MiR-101*, a miRNA commonly down-regulated in cancer, has been implicated in several key cancer-related processes including cell growth, migration, invasion and apoptosis. Recently, several studies supporting the considerable role of *miR-101* in the development of breast cancer have been reported. Sachdeva *et al*. demonstrated that *miR-101* stimulated estrogen-independent growth via upregulation of phosphorylated AKT [28]. Frankel *et al*. revealed that *miR-101* could act as a key regulator of autophagy, which may sensitize breast cancer cells to 4-hydroxytamoxifen (4-OHT)-mediated cell death [30]. Wang *et al*. revealed that *miR-101* was down-regulated in different subtypes of breast cancer, and subsequently showed that *miR-101* could inhibit tumor growth and stimulate breast cancer cells to apoptosis by targeting *STMN1* [31]. To date, only one published association study investigated the effect of *miR-101* polymorphisms on risk of hepatitis B-related liver disease [32]. This study revealed that the rs7536540 polymorphism located in the primary region of *miR-101-1* was significantly decreased the risk of liver cirrhosis and hepatocellular carcinoma (OR = 0.63, 95% CI 0.42–0.93 and OR = 0.63, 95% CI 0.46–0.85 under the dominant model). Furthermore, rs12375841 in *miR-101-2* was significantly associated with clearance of hepatitis B viral infection (OR = 1.24, 95% CI 1.03–1.48 under the co-dominant model). In this study, we did not observe the significant association between the 5 tagging SNPs (rs555146, rs578481, rs705509, rs7536540, rs1011210) in the vicinity of *miR-101-1* gene and the risk of breast cancer. We found that rs462480, a SNP in high LD with rs12375841 (r^2^ = 1) in *miR-101-2*, was significantly associated with an increased risk of breast cancer (OR = 1.182, 95% CI 1.030–1.357 under the additive model). Meanwhile, the SNP rs1053872 in the flanking region of *pre-miR-101-2* was also associated with the susceptibility of breast cancer. In addition, we observed a clear and significant trend toward increased breast cancer risk as the number of variant genotypes of the two SNPs rs462480 and rs1053872.

The pri-miRNA, which is hundreds to thousands of nucleotides in length, is cleaved into pre-miRNA by Drosha in the nucleus, and is subsequently cleaved by Dicer in the cytoplasm to generate the final miRNA duplex [9]. For many pri-miRNAs, RNA folding algorithms have predicted that the sequences flanking either side of the pre-miRNA hairpin, may anneal to form a long, imperfect stem. A modest stem extension adjacent to the pre-miRNA is essential for excision of the pre-miRNA intermediate from a pri-miRNA substrate [33]. Although the function of these extensions or how they regulate the Drosha enzyme remains unclear, the extra flanking sequences may be required initially to tether or recruit the Drosha-DGCR8 complex to RNA [34]. Previous studies have demonstrated that genetic variants in the extensions may affect the Drosha recognition and cleavage [35]–[36]. Therefore, we speculate that the rs462480 at 61 bp downstream of *pre-miR-101-2* may influence the processing of mature miRNA by affecting cleavage of Drosha. Further studies are warranted to investigate the underlying biologic mechanisms for the association of SNPs of *pre-miR-101* and susceptibility to breast cancer.

In conclusion, our results indicated that genetic variants in the vicinity of *pre-miR-101-2* were associated with breast cancer risk in the Chinese population. The rs462480 and rs1053872 SNPs may be considered as candidate genetic markers for the susceptibility to breast cancer in Chinese women. Further studies incorporating diverse populations and functional assays are required to validate and extend these findings.

## Supporting Information

Table S1 Demographic and selected variables in breast cancer and control patients. NOTE: ^a^ T-tests and χ2 tests were used for continuous or categorical variables, respectively; ^b^ ER and PR status information was available in 869 breast cancer cases.
(DOC)

## References

[1] Ferlay J, Shin HR, Bray F, Forman D, Mathers C, et al (2010) Estimates of worldwide burden of cancer in 2008: GLOBOCAN 2008. Int J Cancer 127 (12) 2893–2917. 21351269 10.1002/ijc.25516

[2] Coughlin SS, Ekwueme DU (2009) Breast cancer as a global health concern. Cancer Epidemiology 33 (5) 315–318. 19896917 10.1016/j.canep.2009.10.003

[3] Porter P (2008) “Westernizing” women's risks? Breast cancer in lower-income countries. N Engl J Med 358 (3) 213–216. 18199859 10.1056/NEJMp0708307

[4] Lichtenstein P, Holm NV, Verkasalo PK, Iliadou A, Kaprio J, et al (2000) Environmental and heritable factors in the causation of cancer – analyses of cohorts of twins from Sweden, Denmark, and Finland. N Engl J Med 343 (2) 78–85. 10891514 10.1056/NEJM200007133430201

[5] Thomas G, Jacobs KB, Kraft P, Yeager M, Wacholder S, et al (2009) A multistage genome-wide association study in breast cancer identifies two new risk alleles at 1p11.2 and14q24.1 (RAD51L1). Nat Genet 41 (5) 579–584. 19330030 10.1038/ng.353 PMC2928646

[6] Zheng W, Long J, Gao YT, Li C, Zheng Y, et al (2009) Genome-wide association study identifies a new breast cancer susceptibility locus at 6q25.1. Nat Genet 41 (3) 324–328. 19219042 10.1038/ng.318 PMC2754845

[7] Turnbull C, Ahmed S, Morrison J, Pernet D, Renwick A, et al (2010) Genome-wide association study identifies five new breast cancer susceptibility loci. Nat Genet 42 (6) 504–507. 20453838 10.1038/ng.586 PMC3632836

[8] Long J, Cai Q, Sung H, Shi J, Zhang B, et al (2012) Genome-wide association study in east Asians identifies novel susceptibility loci for breast cancer. PLoS Genet 8 (2) e1002532. 22383897 10.1371/journal.pgen.1002532 PMC3285588

[9] Bartel DP (2004) MicroRNAs: genomics, biogenesis, mechanism, and function. Cell 116 (2) 281–97. 14744438 10.1016/s0092-8674(04)00045-5

[10] Lee Y, Ahn C, Han J, Choi H, Kim J, et al (2003) The nuclear RNase III Drosha initiates microRNA processing. Nature 425 (6956) 415–419. 14508493 10.1038/nature01957

[11] Calin GA, Sevignani C, Dumitru CD, Hyslop T, Noch E, et al (2004) Human microRNA genes are frequently located at fragile sites and genomic regions involved in cancers. Proc Natl Acad Sci USA 101 (9) 2999–3004. 14973191 10.1073/pnas.0307323101 PMC365734

[12] Lu J, Getz G, Miska EA, Alvarez-Saavedra E, Lamb J, et al (2005) MicroRNA expression profiles classify human cancers. Nature 435 (7043) 834–838. 15944708 10.1038/nature03702

[13] Calin GA, Croce CM (2006) MicroRNA signatures in human cancers. Nat Rev Cancer 6 (11) 857–866. 17060945 10.1038/nrc1997

[14] Ryan BM, Robles AI, Harris CC (2010) Genetic variation in microRNA networks: the implications for cancer research. Nat Rev Cancer 10 (6) 523. 10.1038/nrc2867 PMC2950312 20495573

[15] Hu Z, Chen J, Tian T, Zhou X, Gu H, et al (2008) Genetic variants of miRNA sequences and non-small cell lung cancer survival. J Clin Invest 118 (7) 2600–2608. 18521189 10.1172/JCI34934 PMC2402113

[16] Tian T, Shu Y, Chen J, Hu Z, Xu L, et al (2009) A functional genetic variant in microRNA-196a2 is associated with increased susceptibility of lung cancer in Chinese. Cancer Epidemiol Biomarkers Prev 18 (4) 1183–7. 19293314 10.1158/1055-9965.EPI-08-0814

[17] Hu Z, Liang J, Wang Z, Tian T, Zhou X, et al (2009) Common genetic variants in pre-microRNAs were associated with increased risk of breast cancer in Chinese women. Hum Mutat 30: 79–84. 18634034 10.1002/humu.20837

[18] Hoffman AE, Zheng T, Yi C, Leaderer D, Weidhaas J, et al (2009) microRNA miR-196a-2 and breast cancer: a genetic and epigenetic association study and functional analysis. Cancer Res 69 (14) 5970–7. 19567675 10.1158/0008-5472.CAN-09-0236 PMC2716085

[19] Yang Q, Jie Z, Ye S, Li Z, Han Z, et al (2012) Genetic variations in miR-27a gene decrease mature miR-27a level and reduce gastric cancer susceptibility. Oncogene doi: 10.1038/onc.2012.569. [Epub ahead of print] 10.1038/onc.2012.569 23246964

[20] Wang M, Chu H, Li P, Yuan L, Fu G, et al (2012) Genetic variants in miRNAs predict bladder cancer risk and recurrence. Cancer Res 72 (23) 6173–82. 22846912 10.1158/0008-5472.CAN-12-0688

[21] Shi TY, Chen XJ, Zhu ML, Wang MY, He J, et al (2013) A pri-miR-218 variant and risk of cervical carcinoma in Chinese women. BMC Cancer 13: 19. 23320911 10.1186/1471-2407-13-19 PMC3585813

[22] Varambally S, Cao Q, Mani RS, Shankar S, Wang X, et al (2008) Genomic loss of microRNA-101 leads to overexpression of histone methyltransferase EZH2 in cancer. Science 322 (5908) 1695–9. 19008416 10.1126/science.1165395 PMC2684823

[23] Smits M, Nilsson J, Mir SE, van der Stoop PM, Hulleman E, et al (2010) miR-101 is down-regulated in glioblastoma resulting in EZH2-induced proliferation, migration, and angiogenesis. Oncotarget 1 (8) 710–20. 21321380 10.18632/oncotarget.205 PMC3124376

[24] Su H, Yang JR, Xu T, Huang J, Xu L, et al (2009) MicroRNA-101,down-regulated in hepatocellular carcinoma, promotes apoptosis and suppresses tumorigenicity. Cancer Res 69 (3) 1135–42. 19155302 10.1158/0008-5472.CAN-08-2886

[25] Ren G, Baritaki S, Marathe H, Feng J, Park S, et al (2012) Polycomb protein EZH2 regulates tumor invasion via the transcriptional repression of the metastasis suppressor RKIP in breast and prostate cancer. Cancer Res 72 (12) 3091–104. 22505648 10.1158/0008-5472.CAN-11-3546

[26] Carvalho J, van Grieken NC, Pereira PM, Sousa S, Tijssen M, et al (2012) Lack of microRNA-101 causes E-cadherin functional deregulation through EZH2 up-regulation in intestinal gastric cancer. J Pathol 228 (1) 31–44. 22450781 10.1002/path.4032

[27] Wang R, Wang HB, Hao CJ, Cui Y, Han XC, et al (2012) MiR-101 is involved in human breast carcinogenesis by targeting Stathmin1. PLoS One 7 (10) e46173. 23071542 10.1371/journal.pone.0046173 PMC3469601

[28] Sachdeva M, Wu H, Ru P, Hwang L, Trieu V, et al (2011) MicroRNA-101 -mediated Akt activation and estrogen- independent growth. Oncogene 30 (7) 822–31. 20956939 10.1038/onc.2010.463

[29] Qin Z, Xue J, He Y, Ma H, Jin G, et al (2013) Potentially functional polymorphisms in ATG10 are associated with risk of breast cancer in a Chinese population. Gene 527 (2) 491–5. 23850577 10.1016/j.gene.2013.06.067

[30] Frankel LB, Wen J, Lees M, Høyer-Hansen M, Farkas T, et al (2011) microRNA-101 is a potent inhibitor of autophagy. EMBO J 30 (22) 4628–41. 21915098 10.1038/emboj.2011.331 PMC3243595

[31] Wang R, Wang HB, Hao CJ, Cui Y, Han XC, et al (2012) MiR-101 is involved in human breast carcinogenesis by targeting Stathmin1. PLoS One 7 (10) e46173. 23071542 10.1371/journal.pone.0046173 PMC3469601

[32] Bae JS, Kim JH, Pasaje CF, Cheong HS, Lee TH, et al (2012) Association study of genetic variations in microRNAs with the risk of hepatitis B-related liver diseases. Dig Liver Dis 44 (10) 849–54. 22658643 10.1016/j.dld.2012.04.021

[33] Zeng Y, Cullen BR (2005) Efficient processing of primary microRNA hairpins by Drosha requires flanking nonstructured RNA sequences. J Biol Chem 280 (30) 27595–603. 15932881 10.1074/jbc.M504714200

[34] Han J, Lee Y, Yeom KH, Kim YK, Jin H, et al (2004) The Drosha-DGCR8 complex in primary microRNA processing. Genes Dev 18 (24) 3016–27. 15574589 10.1101/gad.1262504 PMC535913

[35] Auyeung VC, Ulitsky I, McGeary SE, Bartel DP (2013) Beyond Secondary Structure: Primary-Sequence Determinants License Pri-miRNA Hairpins for Processing. Cell 152 (4) 844–58. 23415231 10.1016/j.cell.2013.01.031 PMC3707628

[36] Sun G, Yan J, Noltner K, Feng J, Li H, et al (2009) SNPs in human miRNA genes affect biogenesis and function. RNA 15 (9) 1640–51. 19617315 10.1261/rna.1560209 PMC2743066

