# Supplementary material for: Genetic Variations in the Flanking Regions of miR-101-2 Are Associated with Increased Risk of Breast Cancer
Source: PLoS One. 2014 Jan 24;9(1):e86319. doi: 10.1371/journal.pone.0086319 (PMC3901682; doi:10.1371/journal.pone.0086319)
Supplement: Table S1 — Demographic and selected variables in breast cancer and control patients. NOTE: a T-tests and χ2 tests were used for continuous or categorical variables, respectively; b ER and PR status information was available in 869 breast cancer cases. (DOC) [file pone.0086319.s001.doc]

**Table S1. Demographic and selected variables in breast cancer and control patients**

| **Variables** | **Patients** | **Controls** | ***P ^a^*** |
| --- | --- | --- | --- |
|  | **(N=1064)** | **(N=1073)** |  |
| Age, year (mean ± SD) | 50.89±11.44 | 51.51±11.82 | 0.217 |
| Age at menarche, year (mean ± SD) | 15.23±1.93 | 16.11±1.94 | <0.0001 |
| Age at ﬁrst live birth, year (mean ± SD) | 25.57±3.25 | 24.62±3.35 | <0.0001 |
| Age at natural menopause, year (mean ± SD) | 49.72±3.53 | 49.59±3.98 | 0.606 |
| Menopausal status |  |  | <0.0001 |
| Premenopausal | 523 | 505 |  |
| Natural menopause | 451 | 525 |  |
| Unnatural menopause | 78 | 23 |  |
| Estrogen receptor (ER)^b^ |  |  |  |
| Positive | 490 |  |  |
| Negative | 379 |  |  |
| Progesterone receptor (PR)^b^ |  |  |  |
| Positive | 506 |  |  |
| Negative | 363 |  |  |

^a^ T-tests and *χ*2 tests were used for continuous or categorical variables, respectively;

^b^ ER and PR status information was available in 869 breast cancer cases.
